# *Coccidioides posadasii* Infection in Bats, Brazil

**DOI:** 10.3201/eid1804.111641

**Published:** 2012-04

**Authors:** Rossana de Aguiar Cordeiro, Kylvia Rocha de Castro e Silva, Raimunda Sâmia Nogueira Brilhante, Francisco Bergson Pinheiro Moura, Naylê Francelino Holanda Duarte, Francisca Jakelyne de Farias Marques, Rebecca de Aguiar Cordeiro, Renato Evando Moreira Filho, Roberto Wagner Bezerra de Araújo, Tereza de Jesus Pinheiro Gomes Bandeira, Marcos Fábio Gadelha Rocha, José Júlio Costa Sidrim

**Affiliations:** Universidade Federal do Ceará, Fortaleza-Ceará, Brazil (R.A. Cordeiro, K.R.C. Silva, R.S.N. Brilhante, F.J.F. Marques, R.A. Cordeiro, R.E. Moreira Filho, R.W.B. Araújo, T.J.P.G. Bandeira, M.F.G. Rocha, J.J.C. Sidrim);; Instituto Federal de Educação, Ciência e Tecnologia, Ceará, Brazil (K.R.C. Silva); Universidade Estadual do Ceará, Fortaleza- Ceará (M.F.G. Rocha);; Secretaria da Saúde do Estado do Ceará, Ceará (F.B.P. Moura, N.F.H. Duarte)

**Keywords:** Coccidioidomycosis, bats, Carollia perspicillata, fungal eco-epidemiology, endemic mycosis, Histoplasma capsulatum, Coccidioides posadasii, Glossophaga soricina, Desmodus rotundus, fungi, Brazil

## Abstract

To analyze the eco-epidemiologic aspects of *Histoplasma capsulatum* in Brazil, we tested 83 bats for this fungus. Although *H. capsulatum* was not isolated, *Coccidioides posadasii* was recovered from *Carollia perspicillata* bat lungs. Immunologic studies detected coccidioidal antibodies and antigens in *Glossophaga soricina* and *Desmodus rotundus* bats.

Studies have demonstrated that bats (order Chiroptera) are reservoirs for many infectious agents, including protozoa, bacteria, viruses, and fungi (*1*). Several studies confirm that bats have a great effect on human health because they can transmit numerous infectious agents and provide a reservoir for emerging pathogens (*1**,**2*). The interaction between these animals and pathogenic fungi is well illustrated by the occurrence of histoplasmosis outbreaks in humans who are exposed to bat droppings in the environment (*3**,**4*). In Brazil, histoplasmosis is an endemic disease that occurs mainly in patients with AIDS (*5*), but *Histoplasma capsulatum* var. *capsulatum* has also been isolated from bats captured in urban areas (*4*).

To analyze the eco-epidemiologic aspects of *H. capsulatum* in northeast Brazil, we captured bats from urban and rural areas of Ceará State. However, the research revealed the existence of a bat that was naturally infected with *Coccidioides posadasii* and 2 other chiropterans with coccidioidal immunologic responses. This fungal pathogen can cause coccidioidomycosis, a serious infection in humans and animals. The mycosis is presently considered to be endemic to Northeast Brazil, as evidenced by human autochthonous cases (*6**–**8*), positive coccidioidin skin-test results (*7*), and isolation of the fungus from soil (*7**,**9*). We describe the isolation of *C. posadasii* in bats and discuss the epidemiologic effects of this finding.

## The Study

From August 2010 to March 2011, a total of 83 bats of 7 species were captured in 6 cities in Ceará State, Northeast Brazil, where patients with histoplasmosis are seen: Ubajara, Itapiúna, Quixadá, Russas, Aracoiaba, and Baturité. The animals were captured during the day (nonhematophagous bats) or night (hematophagous bats) by using nylon mist nets with 36-mm mesh. The study was part of the rabies control surveillance program headed by the Ceará State Health Department and was approved by the ethics committee of the State University of Ceará (process 07381395–8).

Immediately after capture, the bats were euthanized by an overdose of diethyl ether by inhalation, and their spleen, liver, and lungs were analyzed for *H. capsulatum* isolation. Fragments of each organ were homogenized by maceration in saline supplemented with 200 mg/L chloramphenicol. Aliquots of 100 µL were seeded onto plates containing brain–heart infusion agar, supplemented with 1% glucose, 0.1% l-cysteine, 200 mg/L chloramphenicol, and 0.05% cycloheximide, and incubated at 25°C or 35°C for as long as 6 weeks (*10*). Remaining aliquots of each homogenate, as well as organ fragments, were kept at −20°C.

Although none of the samples were positive for *H. capsulatum*, a colony (Figure) suggestive of *Coccidioides* spp. (Figure, panel A) was isolated from lung homogenates (incubated at 35°C) from *Carollia perspicillata* bats. Microscopic analysis showed hyaline septate hyphae and arthroconidia alternating with empty disjunctor cells (Figure, panel B). Lung fragments from the infected bat were then removed from storage and examined by direct microscopy, revealing coccidioidal spherules (Figure, panel C). The suspected *Coccicioides* colony was evaluated through the in vivo reversion test (*9*). In brief, 5 mL of 0.9% saline was added to a well-sporulating slant culture (15 days old) that was then gently scraped with a cotton swab. Two mice were injected intraperitoneally with 1 mL of the homogeneous supernatant and then were held under biosafety level 3 conditions for 4 weeks. After this period, the animals were euthanized and their spleen, liver, and lungs were removed. Fragments were examined for coccidioidal spherules by direct microscopy with 10% potassium hydroxide and also cultured on BBL Mycosel Agar (Becton, Dickinson and Company, Franklin Lakes, NJ, USA). Additional histopathologic analyses of each organ were performed. Spherules with endospores were found in the lungs of the infected animals (Figure, panel D), and histopathologic analysis supported the identification of *Coccidioides* spp. (Figure, panels E, F). Fragments of the spleen, liver, and lungs cultured on Mycosel Agar yielded mold colonies that produced typical coccidioidal arthroconidia. An additional test was performed by specific PCR reaction (*11*) with Coi9–1F (5′-TACGGTGTAATCCCGATACA-3′) and Coi9–1R (5′-GGTCTGAATGATCTGACGCA-3′) primers to confirm the identity of the pathogen. A voucher of the fungal strain was deposited in the Specialized Medical Mycology Center Culture Collection at the Federal University of Ceará under code CEMM 05–5-059.

**Figure:**
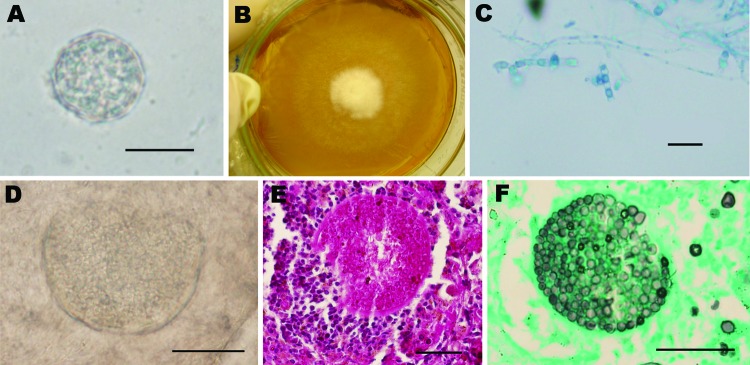
Coccidioidal structures obtained from a naturally infected *Carollia perspicillata* bat (upper images) and experimentally infected mice (lower images). A) Macroscopic aspect of *Coccidioides posadasii* culture recovered from homogenate of bat lungs. B) Microscopic view of *C. posadasii* culture from bat lungs showing hyaline hyphae with arthroconidia and disjunctor cells (lactophenol cotton blue staining). C) Mature spherule filled with endospores in lung tissue (10% KOH) of bat. D) Bursting spherule with endospores in mouse lung tissue (10% KOH). E) Histopathologic features of mouse lungs revealing parasitic coccidioidal forms by periodic acid-Schiff staining. F) Coccidioidal forms on mouse lungs shown by Grocott-Gomori methenamine-silver staining. Scale bars = 20 μm.

Homogenates of lungs, spleen, and liver of all bats were removed from storage and assayed by immunodiffusion tests specific for *H. capsulatum* and *C. posadasii* antigens (*12*) (ID Antigen H & M and IDCF Antigen; Immy Immunodiagnostics, Inc., Norman, OK, USA) according to the manufacturer’s instructions. None of the homogenates showed positive reactions in *H. capsulatum* immunodiffusion tests. However, positive antibodies against *Coccidioides* spp. were found in 1 sample of lung from *Glossophaga soricina* bats. Positive antigen reactions were seen in homogenate liver samples from 2 animals, identified as *G. soricina* and *Desmodus rotundus* bats. These results suggest natural coccidioidal infection among the animals evaluated.

Positive *C. perspicillata* and *G. soricina* bats were captured in the same place, a deserted house in the urban area of Aracoiaba (4°21′59.1′′S and 38°48′51.9′′W) that has a semi-arid climate, with a rainy season from February through April and an average rainfall of 1,010.3 mm per year. The vampire bat, *D. rotundus,* was captured inside a cave in Ubajara (3°48′14.3′′S and 40°52′46.2′′W), a city characterized by a warm, subhumid tropical climate, with a rainy period from January through April and rainfall of 1,483.5 mm per year.

## Conclusions

*Coccidioides* spp. can infect many mammal species (*13*). In this study, *C. posadasii* was isolated from the lungs of *C. perspicillata* bats, a colonial species that can cohabitate with different species of chiropterans (*14**,**15*). We propose 3 hypotheses for this finding. First, we hypothesize that the ability to travel long distances daily in their search for food and the social behavior of chiropterans may promote the acquisition and dispersion of *C. posadasii*. As a result, infected bats may have migrated from areas where *Coccidioides* infections are endemic and introduced the fungus in previously non–disease-endemic areas. A second hypothesis involves the possible existence of other animals that cohabitate with bats in artificial or natural shelters as the primary source of *C. posadasii* infections. Our third hypothesis is that climate changes in recent decades, mainly the increasing temperature in South America, along with the desertification process, which affects approximately one third of Ceará State, might have contributed to this unusual finding. Hypothetical links between climate changes and the epidemiology of other fungal diseases have been described. Studies need to be performed to investigate the role of chiropterans in the epidemiologic cycle of coccidioidomycosis.
